# Pacemaker lead-induced tricuspid regurgitation: consider leaflet remodeling

**DOI:** 10.1007/s10554-020-02131-w

**Published:** 2021-01-27

**Authors:** Federico Fortuni, Frank van der Kley, Victoria Delgado, Nina Ajmone Marsan

**Affiliations:** Department of Cardiology, Heart Lung Center, Leiden University Medical Center, Albinusdreef 2, 2300 RC Leiden, The Netherlands

Cardiac implantable electronic device (CIED) can interfere with tricuspid valve (TV) function, induce significant tricuspid regurgitation (TR), and worsen patient prognosis [1]. We present a case of pacemaker (PM) lead induced severe TR (Fig. 1, Panel A-B) that led to progressive right atrial dilation and heart failure (HF) symptoms 2 years after PM implantation. Transthoracic 2- and 3-dimensional echocardiograms demonstrated a mechanical compression of the TV septal leaflet by the PM lead (arrow on Panel B and D of Fig.1 and Supplemental Video). The patient underwent lead extraction and a leadless PM was positioned at the apex of the right ventricle (RV). Intracardiac echocardiography was performed during the procedure and showed persistence of severe TR after lead extraction (Fig. 1, Panel E) due to retraction (without rupture) of the septal leaflet (asterisk on Panel E of Fig. 1). Transthoracic echocardiography confirmed these findings and showed no significant RV remodeling (Fig. 1, Panel C). Due to persistence of HF symptoms, the patient underwent surgical TV replacement and the intra-operative inspection confirmed an isolated significant thickening and fibrosis of the TV septal leaflet (without rupture) probably induced by the mechanical compression of the PM lead. This case underlines the importance of aiming for a CIED lead position that does not interfere with the TV apparatus to avoid development of TR. Furthermore, we suggest careful imaging and clinical evaluation of patients who develop TR after CIED implantation to optimize the timing for lead repositioning, since TR may not be reversible after lead extraction and surgical correction is needed if irreversible damages of the TV leaflets have occurred.

**Fig. 1:**
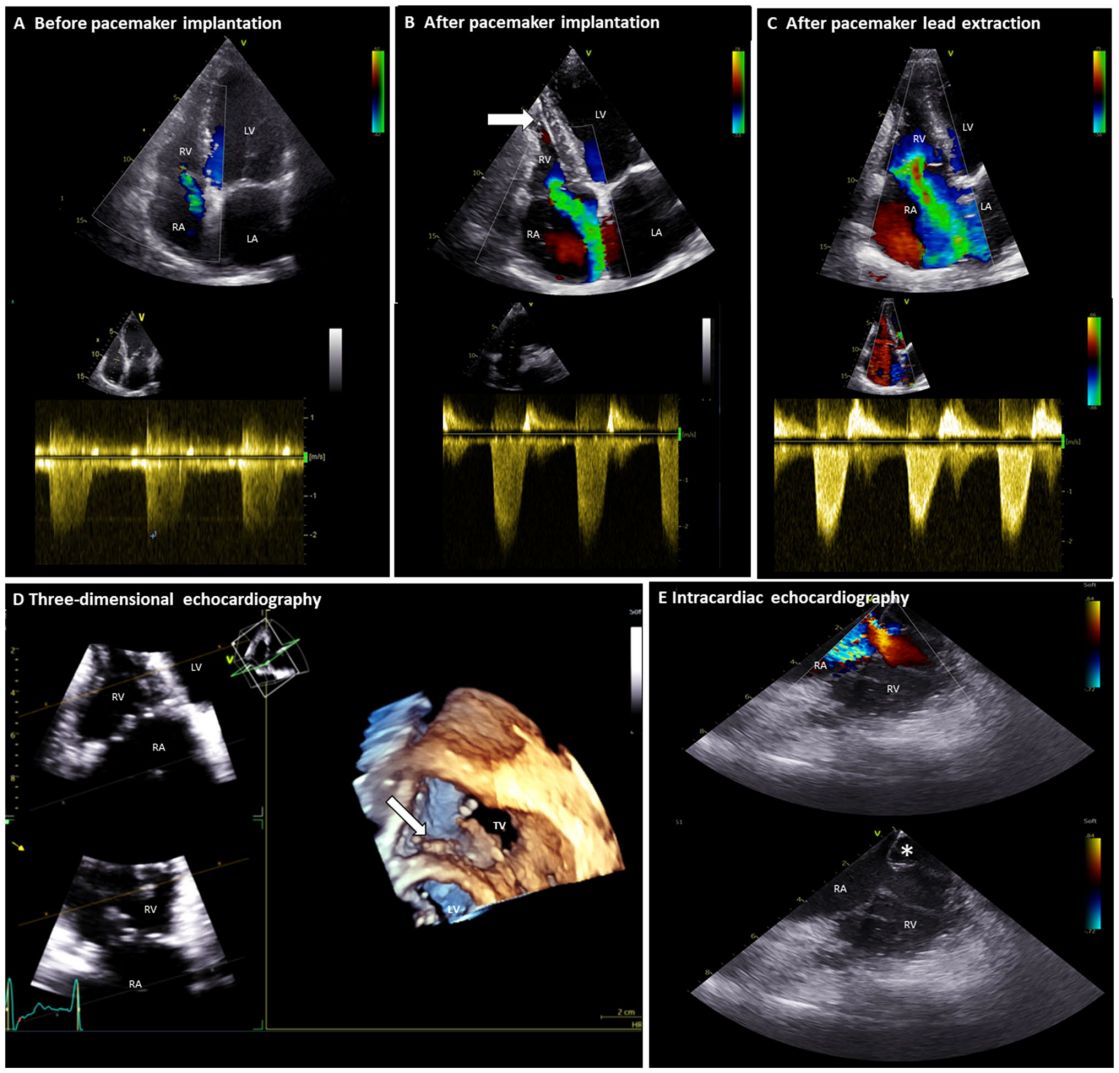
Pacemaker lead-induced tricuspid regurgitation. *LA* left atrium, *LV* left ventricle, *RA* right atrium, *RV* right ventricle, *TV* tricuspid valve

## Supplementary information

Below is the link to the electronic supplementary material.

Supplemental Video (MP4 2869 kb)
